# CBL Is Frequently Altered in Lung Cancers: Its Relationship to Mutations in MET and EGFR Tyrosine Kinases

**DOI:** 10.1371/journal.pone.0008972

**Published:** 2010-01-29

**Authors:** Yi-Hung Carol Tan, Soundararajan Krishnaswamy, Suvobroto Nandi, Rajani Kanteti, Sapana Vora, Kenan Onel, Rifat Hasina, Fang-Yi Lo, Essam El-Hashani, Gustavo Cervantes, Matthew Robinson, Stephen C. Kales, Stanley Lipkowitz, Theodore Karrison, Martin Sattler, Everett E. Vokes, Yi-Ching Wang, Ravi Salgia

**Affiliations:** 1 Department of Life Science, National Taiwan Normal University, Taipei, Taiwan; 2 Department of Medicine, The University of Chicago Cancer Research Center, The University of Chicago Medical Center, Pritzker School of Medicine, Chicago, Illinois, United States of America; 3 Department of Pediatrics, The University of Chicago Cancer Research Center, The University of Chicago Medical Center, Pritzker School of Medicine, Chicago, Illinois, United States of America; 4 Laboratory of Cellular and Molecular Biology, Center for Cancer Research, National Cancer Institute, National Institutes of Health, Bethesda, Maryland, United States of America; 5 Department of Statistics, The University of Chicago, Chicago, Illinois, United States of America; 6 Department of Medical Oncology, Dana-Farber Cancer Institute and Harvard Medical School, Boston, Massachusetts, United States of America; 7 Department of Pharmacology, College of Medicine, National Cheng Kung University, Tainan, Taiwan; City of Hope National Medical Center, United States of America

## Abstract

**Background:**

Non-small cell lung cancer (NSCLC) is a heterogeneous group of disorders with a number of genetic and proteomic alterations. c-CBL is an E3 ubiquitin ligase and adaptor molecule important in normal homeostasis and cancer. We determined the genetic variations of c-*CBL*, relationship to receptor tyrosine kinases (EGFR and MET), and functionality in NSCLC.

**Methods and Findings:**

Using archival formalin-fixed paraffin embedded (FFPE) extracted genomic DNA, we show that c-CBL mutations occur in somatic fashion for lung cancers. c-CBL mutations were not mutually exclusive of MET or EGFR mutations; however they were independent of p53 and KRAS mutations. In normal/tumor pairwise analysis, there was significant loss of heterozygosity (LOH) for the c-*CBL* locus (22%, n = 8/37) and none of these samples revealed any mutation in the remaining copy of c-CBL. The c-*CBL* LOH also positively correlated with EGFR and MET mutations observed in the same samples. Using select c-CBL somatic mutations such as S80N/H94Y, Q249E and W802* (obtained from Caucasian, Taiwanese and African-American samples, respectively) transfected in NSCLC cell lines, there was increased cell viability and cell motility.

**Conclusions:**

Taking the overall mutation rate of c-CBL to be a combination as somatic missense mutation and LOH, it is clear that c-CBL is highly mutated in lung cancers and may play an essential role in lung tumorigenesis and metastasis.

## Introduction

In the US alone, each year approximately 219,400 people are diagnosed with lung cancers, out of which more than 145,000 of them succumb to the disease [1]. This number is roughly equivalent to the combined mortality rates of cancers of the breast, prostate, colon, liver, kidney and melanoma [1]. In addition the prognosis is usually poor and the five-year survival rate is less than 15%. There are also significant ethnic differences for lung cancer, and the outcome is worse for blacks compared to whites. Gender differences are also striking with women having significantly better prognosis as compared to men. There are a number of genetic alterations that can occur in lung cancer. As an example, in NSCLC, mutations in KRAS, p53, EGFR and MET have been identified. Many of these pathways, especially Receptor Tyrosine Kinases (RTKs) are controlled by c-CBL.

*CBL* (Casitas B-lineage lymphoma) is a mammalian gene located on human chromosome 11q23.3 [2] and is involved in cell signaling and protein ubiquitination [3]. CBL proteins belong to the RING finger class of ubiquitin ligases (E3) and there are three homologues *c-CBL*, *CBL-b*, *CBL-3* [4]. The *c-CBL* and *CBL-b* genes are ubiquitously expressed with the highest levels in hematopoietic tissues [5]. *c*-CBL consists of four regions encoding for functionally distinct protein domains: the N-terminal tyrosine kinase binding (TKB) domain, the linker region, the catalytic RING finger domain, the proline-rich region and the c-terminal ubiquitin-associated (UBA) domain that also overlaps with a leucine-zipper (LZ) domain [3]. Both TKB and RING finger domains are essential for ligand-induced ubiquitination of RTKs [6], [7], [8], [9]. The RING finger domain is required for the recruitment of E2 ubiquitin-conjugating enzymes. The TKB domain includes four-helix bundle (4H), a calcium-biding EF hand, and a modified SH2 domain, which binds to phosphotyrosine residues [3], [10], [11], [12]. In addition, the proline-rich region of c-CBL can associate with the SH3 domain of Grb2, which can indirectly recruit c-CBL to RTKs via the GRB2 adaptor protein [7], [13], [14].

c-CBL also binds to EGFR and acts as the E3 that targets EGFR for ubiquitination and degradation. Furthermore, CBL desensitizes EGF signaling and opposes cellular proliferation induced by EGF [15]. EGF activation also appears to activate the tyrosine kinase SRC, which phosphorylates c-CBL and in turn activates the ubiquitination and degradation of EGFR [16], [17], [18]. A recent study shows that defective endocytosis of EGFR is characterized by a deletion mutant and the point mutation L858R, whereby its association with c-CBL and subsequent ubiquitination are impaired [19]. Recently, the first human c-CBL mutations were reported in acute myeloid leukemia (AML) patients [20]. The mutation R420Q inhibits FMS-like tyrosine kinase 3 (FLT3) internalization and ubiquitination [20].

Not only can E3 activity be important in oncogenesis, c-CBL has a dual but separate function as a signal transduction molecule. We have previously shown that c-CBL is important in binding CRKL and BCR/ABL in hematopoietic cells. Also, it can bind and modulate functions of cytoskeleton by binding to proteins like talin and paxillin. The TKB domain is important in binding to a number of molecules, and they then function in signal transduction.

Given the critical role of CBL in normal homeostasis and cancer, we hypothesized that it might be mutated in lung cancers. In this study, we report novel c-CBL somatic mutations S80N/H94Y, Q249E and W802* in Caucasian, Taiwanese and African-American lung cancer patients, respectively. Expressing these mutations in NSCLC cell lines lead to increased proliferation and cell motility. We show that c-CBL mutations occur with or without MET or EGFR mutations but are mutually exclusive of a LOH at the c-*CBL* locus. Additionally, c-*CBL* LOH is associated with either MET or EGFR mutations. We thus hypothesize that c-CBL mutations might contribute to the oncogenic potential of MET and EGFR in lung cancer.

## Methods

### Ethics Statement

Written consent on all research on human subjects has been obtained from the Institutional Review Board, University of Chicago and covers all research performed in the laboratory. The following is their contact information: Institutional Review Board, The University of Chicago, McGiffert Hall, 5751 S. Woodlawn Ave., 2nd floor, Chicago, IL 60637. Written informed consents were received from all patients whose tissue samples were used for this study.

### Tissue Samples

Lung cancer tissue and paired adjacent normal lung tissues were obtained from 50 Caucasian, 29 African-Americans and 40 Taiwanese NSCLC patients who were recruited at the University of Chicago Hospital (Chicago, USA) (Caucasian and African-American patients) and Taipei Veterans General Hospital of Taiwan (Taiwanese patients) after obtaining appropriate Institutional Review Board permission and informed consent from the patients. Out of 119 samples, 77 were men, 38 were women and 4 were unknown with age at diagnosis ranging from 47 to 90 years. In terms of tumor types, 53 were adenocarcinoma, 32 were squamous cell carcinoma and 34 were large cell carcinoma. 49 were stage I, 14 were stage II, 34 were stage III, and 13 were stage IV (**Table S1**).

### Cell Culture

Human non-small cell lung carcinoma cells A549 and H358 were maintained in DMEM and RPMI-1640, respectively. Human embryonic kidney 293T cells were cultured in DMEM. Media were supplemented with 10% fetal bovine serum, 100 units/ml of penicillin, and 100 µg/ml of streptomycin (Invitrogen, Carlsbad, CA). Cells were cultured at 37°C in a humidified incubator containing 5% CO_2_.

### c-*CBL* Gene Mutational Analysis

Exons 2 to 16 of c-*CBL* gene were individually amplified by polymerase chain reaction (PCR). Primers are listed in **Table S2**. PCR conditions were 1 cycle of 95°C for 5 minutes; 35 cycles of 94°C for 30 seconds, 58°C for 30 seconds and 72°C for 2 minutes; and one cycle of 72°C for 10 minutes. PCR products were treated with ExoSAP-IT (USB Corporation, Cleveland, OH) and sequenced by Big-Dye Terminator Chemistry (Applied Biosystems, Foster City, CA). Sequencing was performed on the forward coding strand with confirmation of *c-CBL* alterations performed by sequencing the reverse strand as well. Chromatograms were analyzed for mutations using Mutation Surveyor v2.61 (Softgenetics, State College, PA).

### Plasmid Constructs and Site-Directed Mutagenesis

The wild-type c-*CBL* cDNA insert was subcloned into the pAlterMax expression vector using *Xho*I and *Sal*I restriction enzyme sites (Promega, Madison, WI). Using this parental plasmid pAlterMax-c-*CBL*, the TKB domain double mutation (S80N/H94Y), the point mutation (Q249E), and the C-terminal point mutation W802* of c-*CBL* were created using the following primers: 5′-GCTGGCGCTAAAGAATAACCCACCTTATATCTTAGAC-3′ and 5′-CTACCAGATACCTACCAGTATCTCCGTACTATCTTGTC-3′ for the double mutation S80N/H94Y; 5′-CTTTACCCGACTCTTTGAGCCCTGGTCCTCTTTGC-3′ for Q249E, and 5′-CAGCTCCTCCTTTGGCTGATTGTCTCTGGATGGTGATC-3′ for W802* along with their complementary primers using the QuickChange Site-Directed Mutagenesis XL kit (Stratagene, La Jolla, CA) according to the manufacturer's instructions. The constructs were confirmed for the point mutations by standard DNA sequencing of both strands.

### Loss of Heterozygosity (LOH) Analysis

Five microsatellites on chromosome 11 (3 on 11q at or within 200 kb up or downstream of the c-*CBL* gene and 2 control markers on 11p) were selected for analysis (**Table S3**). Established microsatellite markers and respective primer sequences were selected from the GeneLoc database (http://genecards.weizmann.ac.il/geneloc/index.shtml, Weizmann Institute of Science, Rehovot, Israel). Primers were custom designed and each forward primer was fluorescently labeled at the 5′ end with FAM, PET, NED, or VIC (Applied Biosystems). Primer annealing temperatures and duplex scores were evaluated with NIST Primer Tools (http://yellow.nist.gov:8444/dnaAnalysis/primerToolsPage.do; National Institute of Standards and Technology, Gaithersburg, MD). Primers were verified by performing PCR with control DNA (isolated from TK6 cells) and resolving the products on agarose gels. Bands were visualized with an UV transilluminator. Genomic DNA was extracted from tumor samples and paired normal lung tissue. Primers were grouped into multiplex combinations shown in **Table S4**. Marker D11S929 served as an internal control to check for consistency in PCRs and of peaks from capillary electrophoresis. Multiplex PCRs were carried out in a volume of 10 µL that contained 1 µL genomic DNA (20–50 ng), 0.5 µM of each primer (1.0 µM total for each primer pair), 400 µM dNTPs, 1X PCR buffer containing MgCl_2_, and 0.2 U *Taq* DNA polymerase. PCR was performed on the ABI GeneAmp 9700 PCR System under the following conditions: 5 min at 94°C; 30 cycles of 30 sec at 94°C, 1 min at 60°C, 1 min at 72°C; and 5 min at 72°C. The PCR products were separated by capillary electrophoresis on an ABI 3130XL DNA Analyzer. Chromatograms were analyzed with Peak Scanner 1.0 and GeneMapper 3.7 software (Applied Biosystems) for allelic alterations. The area of the peaks produced by the DNA PCR products was quantified for each allele. The ratio of the allelic areas was calculated for each tumor and paired normal DNA sample. When the qLOH (allelic ratio for the tumor peaks divided by the allelic ratio of paired normal sample) was ≤0.5 or ≥2.0 for c-*CBL* and at least one other 11q marker in at least two separate experiments, the sample was considered as having an allelic imbalance and interpreted as LOH. Samples were evaluated in at least two separate experiments and samples showing prospective LOH at c-*CBL* repeated a third time which included a new control marker at the *BAX* locus (data not shown) on chromosome 19 to verify integrity of sample DNA.

### Transfection of c-*Cbl* Constructs

The A549 cell line was transfected using the Fugene HD (Roche, Nutley, NJ) reagent according to the manufacturer's instructions. Eight µg of plasmid DNA, containing either no insert (empty vector), wild-type c-*CBL*, S80N/H94Y c-*CBL,* Q249E c-*CBL* or W802* *CBL* was used for transfection in a 6-well culture plate. Cells were harvested 48 h after transfection and analyzed for expression.

### c-*CBL* Knockdown

c-*CBL* knockdown was performed using lentiviral transduction using MISSION lentiviral transduction particles (Sigma-Aldrich, St. Louis, MO) as per manufacturer's instructions. Briefly, 1×10^5^ H358 cells/well were seeded in 6-well plates and infected the following day with c-*CBL* lentiviral shRNA constructs. To generate stable c-CBL knockdown cell lines, cells were selected for 2 days with 1 µg/ml puromycin. c-CBL levels were determined using whole cell lysates by immunoblotting with anti-CBL antibody (Santa Cruz Biotechnologies, Santa Cruz, CA).

### Cell Viability Assay

Cells were transfected as described above in the transfection assay. Forty-eight hours after transfection, viability of cells was assessed using Trypan Blue exclusion.

### Wound Healing Assay

A549 cells were seeded in 6-well plates and cultured for 48 h until 100% confluent. The medium was then changed and the cells were transfected as described in the transfection assay. Twelve hours after transfection, a straight scratch was made across the cell layer using a 1 ml pipette tip. The cells were then gently washed with 1× PBS to remove cellular debris and the media was replaced. Photographs were taken of the wound region every 12 h until 48 h.

### Western Blot Analysis

Forty eight hours after transfection, cells were collected and washed twice in 1X PBS, then lysed in ice-cold lysis buffer (0.5M Tris-HCl with pH 7.4, 1.5 M NaCl, 2.5% deoxycholic acid, 10 mM EDTA, 10% NP-40, 0.5 mM DTT, 1 mM phenylmethylsulfonyl fluoride, 5 µg/mL leupeptin, and 10 µg/mL aprotinin) for 5 minutes. The lysate was centrifuged at 13,000 rpm for 20 minutes at 4°C, and protein content of the supernatant was measured. Total cell lysates (50 µg/well) were separated by SDS-PAGE electrophoresis and the gels transferred onto nitrocellulose membranes (Whatman, Piscataway, NJ). Membranes were blocked with 5% non-fat dry milk in phosphate-buffered saline containing Tween-20 (PBST) (1X PBS, 0.1% Tween-20) for 1 h at room temperature and incubated with the appropriate primary antibody at 4°C overnight. Membranes then were washed three times with PBST and probed with appropriate horseradish peroxidase (HRP)-conjugated secondary antibody for 1 h at room temperature. The membranes were again washed three times in PBST and bands were visualized using Western blot chemiluminescence reagent (BioRad, Valencia, CA) on a Chemidoc Gel documentation system (BioRad, Valencia, CA). Antibodies were obtained from Santa Cruz Biotechnologies and used at the following dilutions (c-CBL, 1∶5000; c-MET, 1∶5000; EGFR, 1∶5000; ubiquitin, 1∶1000; HA, 1∶5000 and β-actin, 1∶10,000).

### Flow Cytometry

Cell cycle analysis was carried out by flow cytometry. Approximately 2×10^6^ cells were grown in media containing 10% FBS. Cells were harvested by trypsin/EDTA treatment, washed with 1X PBS three times and fixed with ice-cold 70% ethanol for 2 h. Cells were washed again with cold PBS and stained with a solution containing 25 µg/mL propidium iodide, 200 µg/mL RNase A, and 0.1% Triton X-100 for 30 minutes in the dark. Cell cycle analysis was performed using a Guava PCA-96 flow cytometer (Guava Technologies, Millipore, Billerica, MA).

### Ubiquitin Ligase Activity

293T cells were maintained in culture in DMEM supplemented with 10% FBS and 1% penicillin (100 units/mL) and streptomycin (100 µg/mL) were transfected with 0.2 µg EGFR-pcDNA3 and 2 µg HA-tagged c-*CBL* constructs as indicated using calcium phosphate according to manufacturer's protocol (Profection, Promega, Madison, WI). Twenty-four hours post-transfection, cells were starved overnight in DMEM supplemented with 0.5% FBS, and then treated with or without EGF (100 ng/ml) for 15 min. The cells were collected and washed two times in ice-cold PBS containing 0.2 mM sodium orthovanadate then lysed in ice-cold lysis buffer (10 mM Tris HCl, pH 7.5, 150 mM NaCl, 5 mM EDTA, 1% Triton X100, 10% Glycerol, 2 mM sodium orthovanadate and protease inhibitors). Lysates were cleared of debris by centrifugation at 16,000 g for 10 min at 4°C. EGFR immunoprecipitations were performed on 200 µg of cleared lysate using 250 ng of rabbit-anti-EGFR and Protein A/G Plus Sepharose overnight at 4°C. Precipitations were washed 5 times in lysis buffer before boiling in Laemmli buffer. Elutions were immunoblotted with anti-ubiquitin and EGFR. Twenty micrograms of cleared lysate were immunoblotted for each of the c-*CBL* constructs using anti-HA.

### Statistical Analysis

Mutation rates between different groups were compared using Fisher's exact test. For continuous variables, group comparisons were performed using analysis of variance (ANOVA) followed by Sidak's adjustment for multiple comparisons. Experiments involving repeated measurements over time were analyzed using repeated measures ANOVA with the Greenhouse-Geisser adjustment to the degrees of freedom. Analyses were conducted using STATA (v10.1) software (Stata Corporation, College Station, TX).

## Results

### c-*CBL* Gene Mutations in Lung Cancer

To investigate the role of c-*CBL* in lung cancer, we analyzed its genomic DNA in tumor and paired normal samples drawn from multiple ethnicities. The lung tumor samples represented Caucasians (n = 50), African-Americans (n = 29), and Taiwanese (n = 40) lung cancer patients. We designed 12 pairs of primers to sequence the coding region of c-*CBL* gene that spans exons 2 to 16 (**Table S2**). We identified 8 unique somatic mutations in c-*CBL exons* among 8 different patients. A variation L620F, a known SNP (rs2227988) in exon 11 was also detected. Importantly, the eight novel non-synonymous mutations were confirmed by sequencing both strands of c-*CBL* genomic DNA obtained from lung tumor samples (**Table 1**). Moreover, none of the 8 mutations were detected in the corresponding normal tissue, indicating that these were somatic mutations. Four synonymous single nucleotide variations (SNVs) were also identified but were not used further in this study.

**Table 1:** c-*CBL* mutation analysis in 119 lung cancer patient tumor tissues.

Location	Mutation	Domain	Numbers of Sample (Frequency)		
			Caucasian (50)*	African-American (29)*	Taiwanese (40)*
**Exon 2**	26354 G>AG **(S80N)^#^**	TKB	1 (2%)	0	0
	26395 C>CT **(H94Y)^#^**	TKB	1 (2%)	0	0
**Exon 4**	67885 C>CG **(Q249E)**	TKB	0	0	1 (2.5%)
**Exon 8**	72104 G>AG **(V391I)**	RING	1 (2%)	1 (3.5%)	0
**Exon 9**	72515_72517 del ATG	Pro-rich	1 (2%)	0	0
**Exon 11**	79346 C>CT **(L620F)** ∧	Pro-rich	0	0	2 (5%)
**Exon 15**	92375 G>AG (W802*)	C-terminal	0	1 (3.5%)	0
**Exon 16**	93412 G>AG **(R830K)**	C-terminal	0	1 (3.5%)	0
	93465 G>AG **(A848T)**	C-terminal	0	1 (3.5%)	0

*( ) indicates number of samples.

**# S80N and H94Y mutations were found in the same patient.**

**∧Known SNP.**

**Figure 1:**
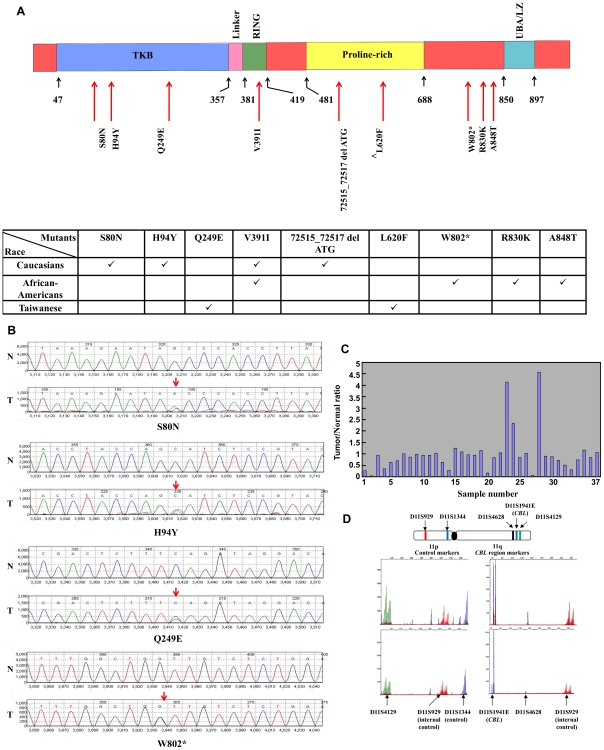
c-*CBL* mutations and LOH in non-small cell lung cancer. (A) Schematic illustration of the various c-CBL mutants with respect to the different functional domains. Three mutations: S80N, H94Y, Q249E were located on the TKB domain; V391I was located on the RING finger domain; 72515_72517 del ATG (a non-frameshift deletion) and L620F (a known SNP, rs2227988) were located on Pro-rich region and W802*, R830K, and A848T were found in the C-terminal region of c-CBL. The numbers shown represent amino acid positions. Association of c-CBL mutations among different ethnic populations is shown in the table (∧ known SNP). (B) Representative examples of sequencing chromatograms of the mutation region in normal (N) and tumor (T) samples. Arrows indicate the heterozygous mutation in the tumor sample. (C) Loss of Heterozygosity (LOH) analysis of 37 tumor and paired normal samples from Taiwanese patients. A value of <0.5 indicates a LOH at the c-*CBL* locus. (D) Schematic of Chromosome 11 markers chosen for LOH analysis and representative examples of LOH chromatogram analysis. After amplification using chromosome 11 specific microsatellite primers, the PCR product was separated by capillary electrophoresis and bands were quantified according to the intensity.

Three of the 8 novel non-synonymous mutations were located in the TKB (tyrosine kinase binding) domain (S80N, H94Y, and Q249E), one in the RING finger domain (V391I), one in the proline-rich region (72515_72517 del ATG), and three in the C-terminal region (W802*, R830K, and A848T) of the c-CBL protein (**Figure 1A** and **Figure S1**). In **Figure 1B**, we show model chromatograms of representative samples.

### 11q LOH of c-*CBL* Gene

Paired lung tumor and normal lung tissue samples from Taiwanese patients (n = 37) were investigated for LOH. Eight (21.6%) showed LOH at the c-*CBL* locus on chromosome 11 while 29 samples (78.4%) revealed normal allelic contribution at the microsatellite markers (**Figures 1C and D**).

### c-CBL Mutations in Different Ethnic Groups

The c-CBL double mutant S80N/H94Y was found in the same patient and the overall mutation rate for c-CBL in lung tumors was 6.7% (8/119). The frequency of c-CBL mutation was highest in large cell carcinoma (14.7%; 5 of 34 patients) followed by squamous carcinoma (6.3%; 2 of 32 patients) and the least was observed in adenocarcinoma (AD) (1.8%; 1 of 53 patients), although these rates were not statistically significant (p = 0.292). Mutation rates were 6.0% among Caucasians (0 of 20 in AD; 0 of 10 in SQ; and 3 of 20 in LC), 13.8% in African-Americans (1 of 10 in AD; 1 of 10 in SQ; and 2 of 9 in LC), and 2.5% (0 of 23 in AD; 1 of 12 in SQ; and 0 of 5 in LC) in the Taiwanese population. Additionally two Taiwanese patients with lung cancer (one squamous and one adenocarcinoma) had the known SNP L620F. Ethnic differences were not statistically significant, however the power to detect differences was low.

### Mutations in *MET* and *EGFR* Can Be Co-Associated with c-CBL Alterations

**Figure 2:**
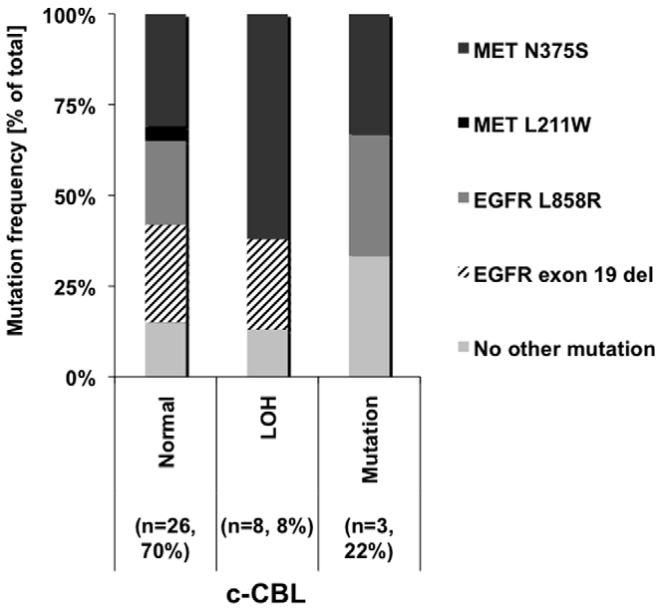
c-*CBL* mutations and relationship to *MET* and *EGFR* mutations in lung cancer. 37 Taiwanese samples were analyzed for mutations in c-*CBL*, *MET* and *EGFR* and for LOH analysis in the c-*CBL* locus. Data shows 21.6% (8/37) had LOH, whereas 8% (3/37) had a c-CBL mutation (including the known SNP L620F), these samples were mutually exclusive. Additionally, 5 of 8 LOH samples also had MET N375S mutation and 2 had *EGFR* exon 19 deletion. In the samples having c-*CBL* mutation, 1 also had MET N375S mutation while another had EGFR L858R mutation. In samples that did not harbor any c-*CBL* mutation (70%, 26/37), 22 had either a *MET* or an *EGFR* mutation and only 4 did not have a mutation in any of these 3 genes.

Since East Asians with lung cancer have a higher frequency of *EGFR* and *MET* mutations in lung tumors [21], [22], we also determined mutations in *EGFR* and *MET* in the same Taiwanese cohort samples and compared the results with the observed c-*CBL* alterations (LOH and/or mutations). In the 37 samples tested, we did not find any overlap between c-*CBL* mutations and c-*CBL* LOH **(** **Figure 2** **)**. Of the three c-CBL mutants (including the known L620F SNP, rs2227988), one of the samples had a MET mutation (N375S) and the other had an EGFR mutation (L858R). Among the 8 samples that had a LOH at the c-*CBL* locus 5 had an additional mutation in MET (N375S) and 2 had an *EGFR* exon 19 deletion. Twenty-six samples had neither c-*CBL* mutation nor c-*CBL* LOH (3 patients had a c-*CBL* mutation but no c-*CBL* LOH). Among these 26 samples 9 had a MET mutation (8 N375S, 1 L211W), 13 had an *EGFR* mutation (7 exon 9 deletion, 6 L858R) and 4 had no other MET or EGFR mutation. Thus the rate of MET or EGFR mutations among patients with LOH at the c-*CBL* locus (7 of 8) was similar to that seen in patients without c-*CBL* mutation or LOH (22 of 26 patients) (p = 0.99). These 4 patients with no identifiable mutation in c-*CBL*, *MET* or *EGFR* represented 10.8% of the 37 patients analyzed in the Taiwanese patient cohort. Conversely, 89.2% Taiwanese lung cancer patients have an identifiable mutation in either c-*CBL*, *MET* or *EGFR* or a combination of the three genes **(** **Figure 2** **)**. Additionally, we determined *p53* and *KRAS* mutations in these Taiwanese cohorts. Two *p53* and 1 *KRAS* mutation were detected. The single *KRAS* mutation overlapped with one *p53* mutation. This patient also had the *EGFR* exon 19 deletion but had no c-*CBL* mutation. The other *p53* mutation sample had a c-*CBL* LOH with concurrent MET N375S mutation. Thus, in the Taiwanese samples analyzed, *p53/KRAS* mutations and c-*CBL* mutations were mutually exclusive (data not shown).

### Cellular Functions of c-*CBL* Alterations in the Context of Lung Tumorigenesis

#### A. E3 activity is intact in the mutant c-CBL proteins

**Figure 3:**
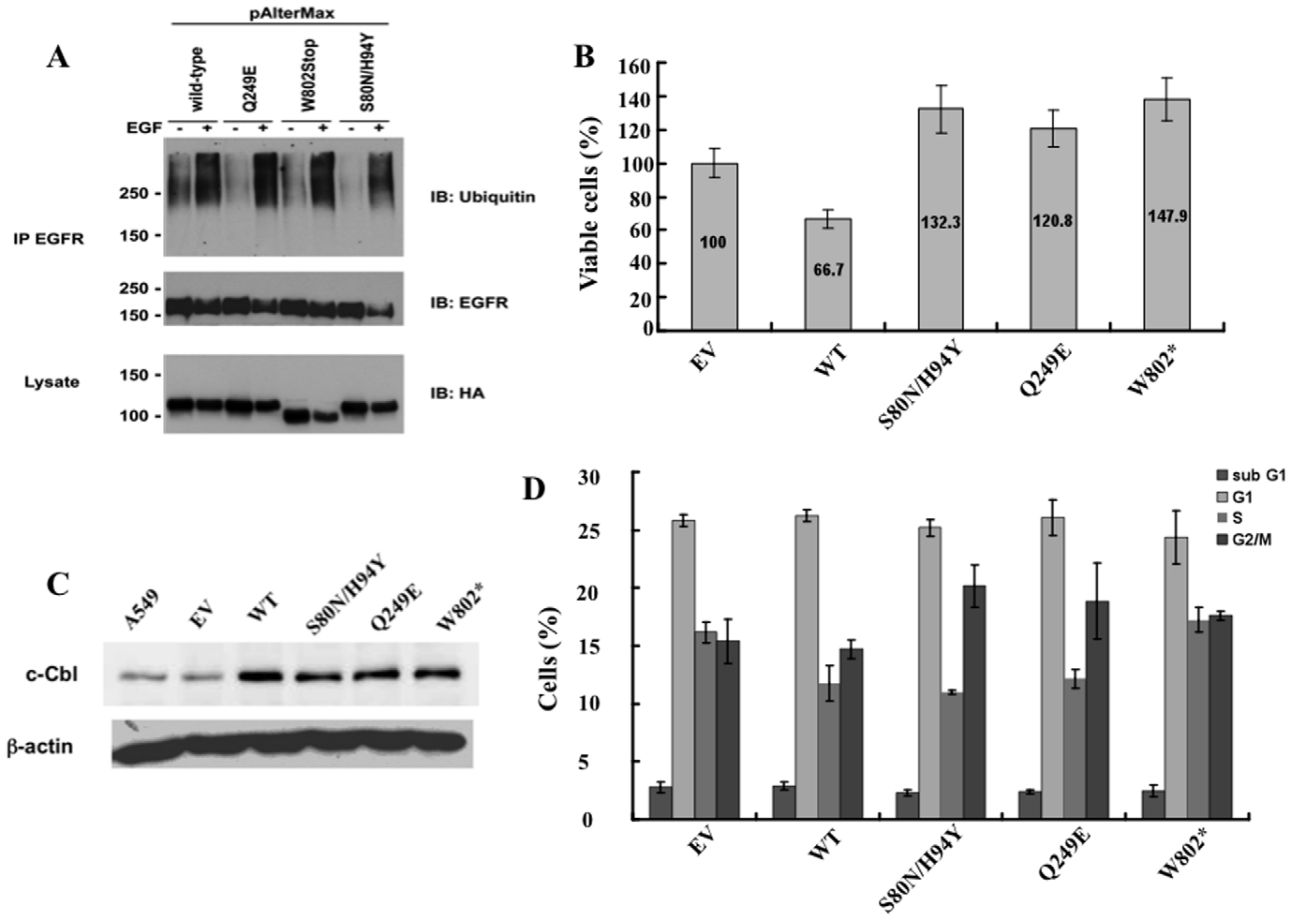
Ubiquitination, viability, expression and cell cycle analysis of various c-CBL mutants. (A) c-CBL mutants do not alter ubiquitination of EGFR. Cells were co-transfected with EGFR and different c-CBL mutants were stimulated with EGF, immunoprecipitated with anti-EGFR antibody and blotted with anti-ubiquitin antibody. Immunoblot with anti-EGFR antibody served as the IP control, while the anti-HA blot was used as the input control. (B) Cell viability was measured by Trypan blue exclusion and compared to empty vector control. c-CBL wild-type (WT) and mutants S80N/H94Y, Q249E, and W802* showed 66.7%, 132.3%, 120.8%, and 147.9% cell viability respectively in A549 cells 48h after transfection. Experiments were done in triplicates and the mean data is shown. Error bars indicate Standard Deviation. (C) Protein expression levels of the various mutants were analyzed by Western blots using c-CBL antibody. (D) Cell cycle analysis of different c-CBL mutants 48 h after transfection in A549 cells.

To investigate whether the different c-CBL mutations affect the E3 activity, EGFR was chosen as a model substrate for c-CBL E3 function. All of the c-CBL mutants tested enhanced ubiquitination of the activated EGFR similar to the wild-type c-CBL protein. This result demonstrates that the catalytic activity of the c-CBL mutants is not impaired when EGFR was the substrate. (**Figure 3A**).

#### B. Effect on lung cancer cell viability

The effect of a representative c-CBL mutant from each of the three ethnic backgrounds on lung cancer cell viability in cell lines was determined. S80N/H94Y double mutation, Q249E, and W802* were identified in lung tumor samples obtained from a Caucasian, a Taiwanese and an African-American, respectively. As described in methods, the c-CBL wild-type (WT) and the above three mutants were expressed after cloning them into pAlterMax vector in A549 cells. These cells express relatively low basal levels of endogenous c-CBL (data not shown). Transfection efficiency was comparable between different groups and the number of cells transfected with c-*CBL* wild-type construct was about 70% compared to control cells that were transfected with the empty vector. Cells transfected with S80N/H94Y, Q249E and W802* c-CBL mutant constructs resulted in increased number of viable cells that was 132.3%, 120.8% and 147.9% higher respectively, relative to the empty vector control transfected cells and significantly different from the wild-type construct (p = 0.022, p = 0.049, and p = 0.008, respectively) (**Figure 3B**). Relative levels of c-CBL protein in whole cell lysates prepared from samples obtained from a parallel experiment was determined. The c-CBL protein levels in samples representing untransfected and empty vector transfected cells were comparable and those representing the c-CBL WT and the three c-CBL mutants were comparable **(** **Figure 3C** **)**.

#### C. Effect on cell cycle

To investigate if the increases in cell viability in different c-CBL mutants are due to increased cellular proliferation, a cell cycle analysis was performed. A549 cells were transfected with the c-CBL WT or the three different mutants: S80N/H94Y, Q249E and W802*. The empty vector transfectant was used as a control. Forty-eight hours after transfection cell cycle analysis was performed as described in materials and methods. There was no significant change in the subG1, G1 or the S phase of the cell cycle among the different mutants compared to the WT construct (p = 0.64, p = 0.40, and p = 0.28, respectively). The G2/M phase of the cell cycle showed an increase in cell numbers for the three mutants, S80N/H94Y, Q249E and W802*, when compared to the WT but again the difference was not statistically significant (p = 0.25) (**Figure 3D**).

#### D. Effect on cell motility

**Figure 4:**
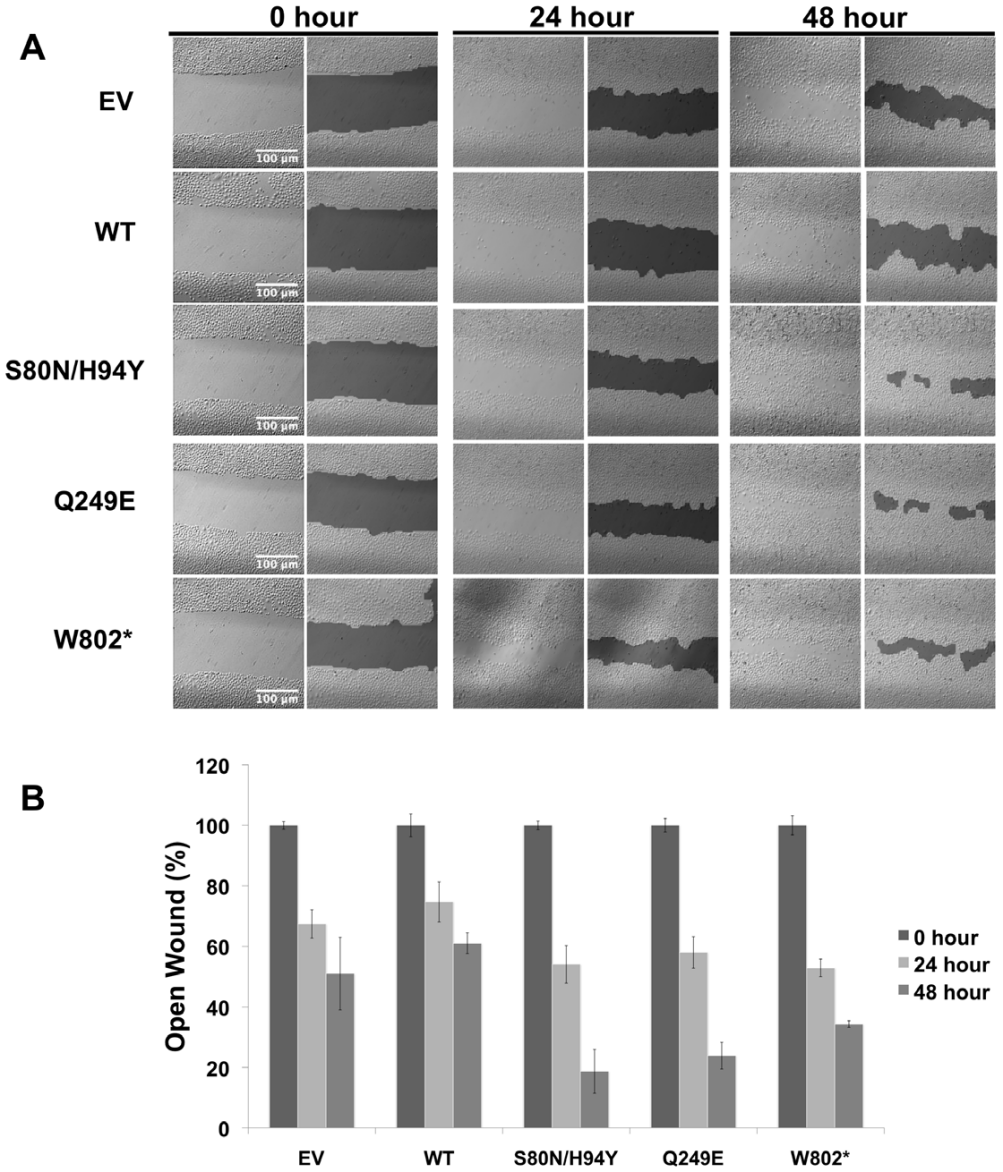
c-CBL mutations affect wound healing in A549 cells. (A) Wound healing assay was performed in A549 cells transfected with WT c-CBL or different mutants. Empty vector (EV) was used as a control. Representative pictures (Brightfield and phase contrast) from each timepoint are shown. (B) The open wound at each time point was quantified and normalized to 0 h. Experiments were done in triplicate and the mean data is shown. Error bars indicate Standard Deviation.

To investigate the effect of the expression of the above three c-CBL mutants on cell migration, we carried out wound healing assay as described in materials and methods. The closing of the scratch or the wound was monitored at 0, 12, 24, 36, and 48 h. (**Figure 4A**). In all the samples, that represented cells transfected with mutants, the wound gap was much smaller than that seen in the sample that represented cells transfected with c-CBL WT (p<0.001). We also determined the rate of wound closure for all the five groups. At 48 h, wild-type c-CBL transfectants showed 61.1% open wound while the S80N/H94Y, Q249E and W802* mutants showed 18.7%, 23.9% and 34.3% open wound respectively (p<0.001) (**Figure 4B**).

#### E. c-CBL knockdown increases cell viability

**Figure 5:**
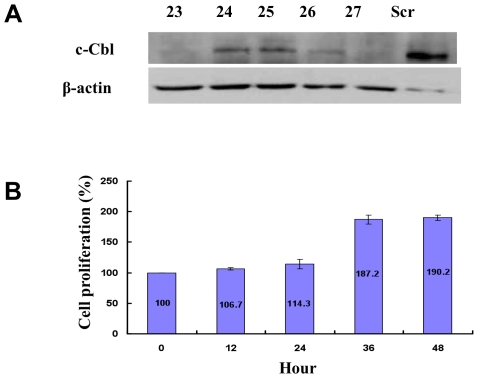
Knockdown of c-*CBL* using an shRNA increases cell proliferation. (A) Western blots of different c-*CBL* shRNA constructs showing knockdown efficiency of different clones. Scrambled shRNA was used as a control. (B) Lung cancer cell line H358 stably transfected with shRNA clone 27 showed an increase in cell counts compared to the scrambled shRNA control. Experiments were done in triplicates and the mean data is shown. Error bars indicate Standard Deviation.

It is hypothesized that the LOH seen in our samples could lead to decreased expression of c-CBL. Thus we tested the effect of c-CBL knockdown in lung cancer cells. Compared to A549, H358 lung cancer cells express relatively high levels of endogenous c-CBL (data not shown). c-CBL expression was knocked down using lentiviral construct that expressed c-*CBL* specific shRNA and compared the results with those that were transduced with scrambled shRNA and the results are shown in **Figure 5**. We identified several clones that revealed varying degrees of c-CBL knockdown showing different sets of c-*CBL* lentiviral shRNA knockdown efficiency (**Figure 5A**). Of all the clones tested, Clone 27 was chosen for further experiments. Equal amount of cells were seeded in a 6-well plate and the cell proliferation was measured at various times and the results are depicted in **Figure 5B**. As expected, number of cells increased in a time dependent fashion from 100 to 190% relative to scrambled shRNA as control in a span of 48 h (p = 0.0002) (**Figure 5B**). The cell cycle phases in H358 cells that were knocked down with c-*CBL* shRNA were looked at and compared with the scrambled shRNA. There were no discernable differences between these two constructs in the different phases of the cell cycle (data not shown).

## Discussion

Our results demonstrate that c-*CBL* is somatically mutated (or has LOH) in lung cancers, and can significantly contribute to enhanced cell viability and motility. There was also a high prevalence of LOH with respect to c-*CBL* in lung tumors that harbored *MET* or *EGFR* mutation.

In the present study, we have demonstrated the occurrence of c-CBL mutations in lung cancer patients, especially with different ancestral variations. Mutations in c-CBL have been recently reported in juvenile myelomonocytic leukemia and myeloid malignancies. In the AML study, the mutation R420Q located in the junction of the RING finger and the linker region inhibited FMS-like tyrosine kinase 3 (FLT3) internalization and ubiquitination [20], thus contributing to the gain-in-function for the RTK. In addition, mutations such as H398Y, C384R, and L380P mapped to the RING finger domain and the linker region of c-CBL that is required for its E3 activity [23], [24], [25], [26], [27]. Additionally, homozygous mutations in the RING finger domain of the c-*CBL* gene were described as a result of acquired Uniparental Disomy (UPD) [26]. It is important to note that our results indicate LOH at 11q23 locus and these are mutually exclusive from missense mutations of c-*CBL*. The somatic mutations were all heterozygous. The mutations in AML led to abrogation of the E3 activity leading to prolonged RTK activation. In addition mutants located on the linker region surrounding the RING finger domain exhibited enhanced AKT signaling in response to cytokine stimulation [26]. In addition, it was shown in NH3T3 cells, that neither mutations in the RING finger nor the linker region causes transformation, however while certain mutations perturbs the ubiquitination, others affect receptor recycling and prolong kinase activity [28].

We report here c-CBL mutations that mapped not only to the RING finger domain, but also to the TKB domain, proline-rich domain and the C-terminal region, but none mapped to the linker region as reported in the AML studies described above [23], [24], [25], [26], [29]. In addition, 8 mutants that we detected were found in different ethnic backgrounds. For example, S80N/H94Y, Q249E, W802* were detected in Caucasians, Taiwanese and African-Americans, respectively. The results point out not only the difference between lung cancer and other cancers, but also genetic polymorphism among different races in the same cancer. Interestingly, there is a large disparity between African-American and other ethnic populations with lung cancer [30]. We have previously shown that there was a low frequency of EGFR and MET mutation in African-Americans as compared with Taiwanese and Caucasians [31]. In this study, the number of African-American samples analyzed was relatively fewer and we found 3 mutations that are unique to this ethnicity. It would now behoove us to further study the genetic alterations that can occur and determine the targeted therapeutics for African-Americans. Our results provide evidence of the importance of c-CBL in tumorigenesis and potential signaling. Our prediction, based on the AML data, would be that the V391I RING finger domain mutation would affect the E3 activity. Also, it will be important to determine the binding partners for c-CBL in the TKB domain and proline rich domain mutations. It has previously been shown that the TKB domain can bind to growth factor receptors and it will be important to determine the cross-binding of these mutants to MET and or EGFR. It would also be important in the future to look at fluorescence in situ hybridization/copy number changes in c-*CBL* in lung cancer.

c-CBL plays an important role in down regulating RTK-mediated signaling through K63 poly-ubiquitination and subsequent downregulation of RTKs followed by lysosomal degredation [3]. Mono-ubiquitination or ubiquitinated with K63-linked chains of substrates by c-CBL may lead to enhancement of biological and biochemical functions (reviewed in Hermann et al, 2007 [32]). The mutations that we analyzed in our studies all point out to the fact that the E3 activity of c-CBL on EGFR is intact; the EGFR levels in the various mutants remain same (**Figure S2**). Multiple kinases, both RTKs and non-RTKs could be acted upon by c-CBL, including ERBs, PDGFR, FMS, MET, c-Kit, VEGFR, FLT-1, RON, FGFR, IR, as well as SYK, FYN, LCK, FGR, LYN and c-ABL [3]. In lung cancers the relevant substrates of c-CBL in terms of degradation or signal transduction are yet to be identified.

The observation that c-CBL somatic mutations, especially S80N/H94Y, Q249E and W802* showed increased cell viability and cell motility that is in agreement with the physiological role for D-cbl in the regulation of apoptosis and differentiation identified in Drosophila is very significant [33]. It has been previously shown that activating c-CBL mutation downregulates EGFR signaling and decreases cellular proliferation and migration in breast cancer cell lines [34]. Although the role of c-CBL in the negative regulation of RTKs is well substantiated, thereby suggesting that it is a natural tumor suppressor, studies in cancer cells have revealed both tumor suppressor and tumor promoting activities depending on the type of c-CBL mutation and the number of alleles at the c-*CBL* locus [24]. In agreement with the above, the three c-CBL mutants described here appear to have tumor growth and metastasis promoting properties. Although these mutants are outside of the RING finger or the linker region of c-CBL, their downstream effects are significant so as to cause increased proliferation and migration, but the substrate affected by these mutations are not known yet. This raises the possibility that some of the cellular functions of c-CBL are independent of its ubiquitin-ligase activity, an area that we are currently investigating. The oncogenic nature of RTKs, addiction of cancers to growth signals and given the clustering of c-*CBL*, *EGFR* and *MET* mutations, it is possible that the transforming effect of c-CBL mutations is most likely a combinatorial effect of the three. We also show that LOH for c-*CBL* was found in a significant number of samples that harbored *MET* or *EGFR* mutations. The fact that about 7% of lung tumor samples are likely to have c-CBL mutations and an additional 22% are likely to harbor c-*CBL*-related LOH, makes c-CBL a highly mutated molecule in lung cancer. Since LOH alone is not enough to cause a transforming event [35], [36], [37], associated mutation in the *MET* or *EGFR* locus or yet another RTK discussed above may play a role in carcinogenesis. We predict that this LOH in c-*CBL* results in haploinsufficiency that downplays RTK ubiquitination leading to hyperactivity of the RTKs. However, whether this is sufficient cause for tumorigenesis remains to be determined. Consistent with our hypothesis is the fact that *c-cbl^-/-^* mice have increased kinase activity in lymphocytes, but is not sufficient for tumor formation [35], [36], [37]. c-*CBL* LOH could also lead to increased expression of c-CBL from the other allele to compensate for a loss of an allele. Alternately, there could be some form of synergy working with reduced c-CBL levels and mutated receptors that exacerbate the phenotype of each alone.

Previous studies from our lab and others have shown that East Asians with lung cancers have relatively high frequencies of gain-of-function of mutations in RTKs such as EGFR and MET [31]. In a cohort of Japanese patients an activating MET mutation was identified in the splice region that deletes the juxtamembrane domain that is involved E3 activity of c-CBL [38]. This study also found that activation of MET is mutually exclusive of EGFR, KRAS and HER2 gene mutations [38]. We failed to detect such mutations in significant numbers in lung tumor samples obtained from African-Americans (n = 29) and Caucasian (n = 50) patients. One MET mutation was identified in each of the groups whereas 1 and 3 EGFR mutations were identified in the African-American and Caucasian cohorts respectively. EGFR mutations have earlier been identified as one of the key mutations affecting lung adenocarcinoma patients in a comprehensive study of 188 patients [39]. Our study encompasses different histologies of NSCLC. However, the published series did not find any mutations in c-*CBL* or *MET* unlike our study that encompassed different subtypes of NSCLC. It is important to note, we have recently shown that MET mutations in lung cancer are in majority germline [31]. We have reported earlier c-CBL mutations in a small cohort of Taiwanese lung cancer samples [40]. In our efforts to understand the ethnic differences in the lung oncogenome, we also looked at PAX transcription factors such as PAX5 and PAX8 that are highly expressed in lung cancers; however there was no preferential expression or mutations of the above genes in lung tumor samples of African-Americans. In this study, we show relatively high frequency of c-CBL mutations in lung cancers, especially in the large cell type among Caucasians and particularly among African-Americans. We therefore propose c-CBL as an efficacious target for lung cancers in African-Americans that needs to be further substantiated. This is all the more important because the prognosis for African-Americans with lung cancer, especially for men is much poorer compared to their Caucasian counterparts [41].

In conclusion, the results presented in this study demonstrate that c-CBL is frequently mutated or even lost in lung cancers. Our results support a role for c-CBL mutants that are independent of its ubiquitination activity. Given the relatively high mutation rates in c-CBL as well as RTKs such as MET and EGFR, it is likely that their combined effect could be synergistic in promoting tumorigenesis.

## Supporting Information

Figure S1 Schematic of c-CBL domains and associated exons. Numbers at the top indicate amino acid residues flanking the domains.
(0.42 MB TIF)

Figure S2 Western blot showing EGFR levels in different c-CBL mutants used in the study. EGFR levels were relatively unchanged among the three mutants compared to wild-type c-CBL. β-actin was used as a loading control.
(3.00 MB TIF)

Table S1 Number of samples analyzed in patients by race and sub-type of lung cancer.
(0.03 MB DOC)

Table S2 c-Cbl PCR amplification primers.
(0.04 MB DOC)

Table S3 Microsatellites and primer information.
(0.03 MB DOC)

Table S4 Multiplex primer sets for LOH analysis.
(0.03 MB DOC)
