# Correction: CBL Is Frequently Altered in Lung Cancers: Its Relationship to Mutations in MET and EGFR Tyrosine Kinases

**DOI:** 10.1371/annotation/940edb14-522e-4f38-b6c4-7557e9a13d15

**Published:** 2011-01-07

**Authors:** Yi-Hung Carol Tan, Soundararajan Krishnaswamy, Suvobroto Nandi, Rajani Kanteti, Sapana Vora, Kenan Onel, Rifat Hasina, Fang-Yi Lo, Essam El-Hashani, Gustavo Cervantes, Matthew Robinson, Han-Shui Hsu, Stephen C. Kales, Stanley Lipkowitz, Theodore Karrison, Martin Sattler, Everett E. Vokes, Yi-Ching Wang, Ravi Salgia

The authors wish to add Dr. Han-Shui Hsu as an author to this paper. The corrected byline is: Yi-Hung Carol Tan, Soundararajan Krishnaswamy, Suvobroto Nandi, Rajani Kanteti, Sapana Vora, Kenan Onel, Rifat Hasina, Fang-Yi Lo, Essam El-Hashani, Gustavo Cervantes, Matthew Robinson, Han-Shui Hsu, Stephen C Kales, Stanley Lipkowitz, Theodore Karrison, Martin Sattler, Everett E Vokes, Yi-Ching Wang, Ravi Salgia. Dr. Hsu's affiliation is: Institute of Emergency and Critical Care Medicine, National Yang-Ming University School of Medicine, Taipei, Taiwan.

