# Supplementary figures and images for: CBL Is Frequently Altered in Lung Cancers: Its Relationship to Mutations in MET and EGFR Tyrosine Kinases
Source: PLoS One. 2010 Jan 29;5(1):e8972. doi: 10.1371/journal.pone.0008972 (PMC2813301; doi:10.1371/journal.pone.0008972)

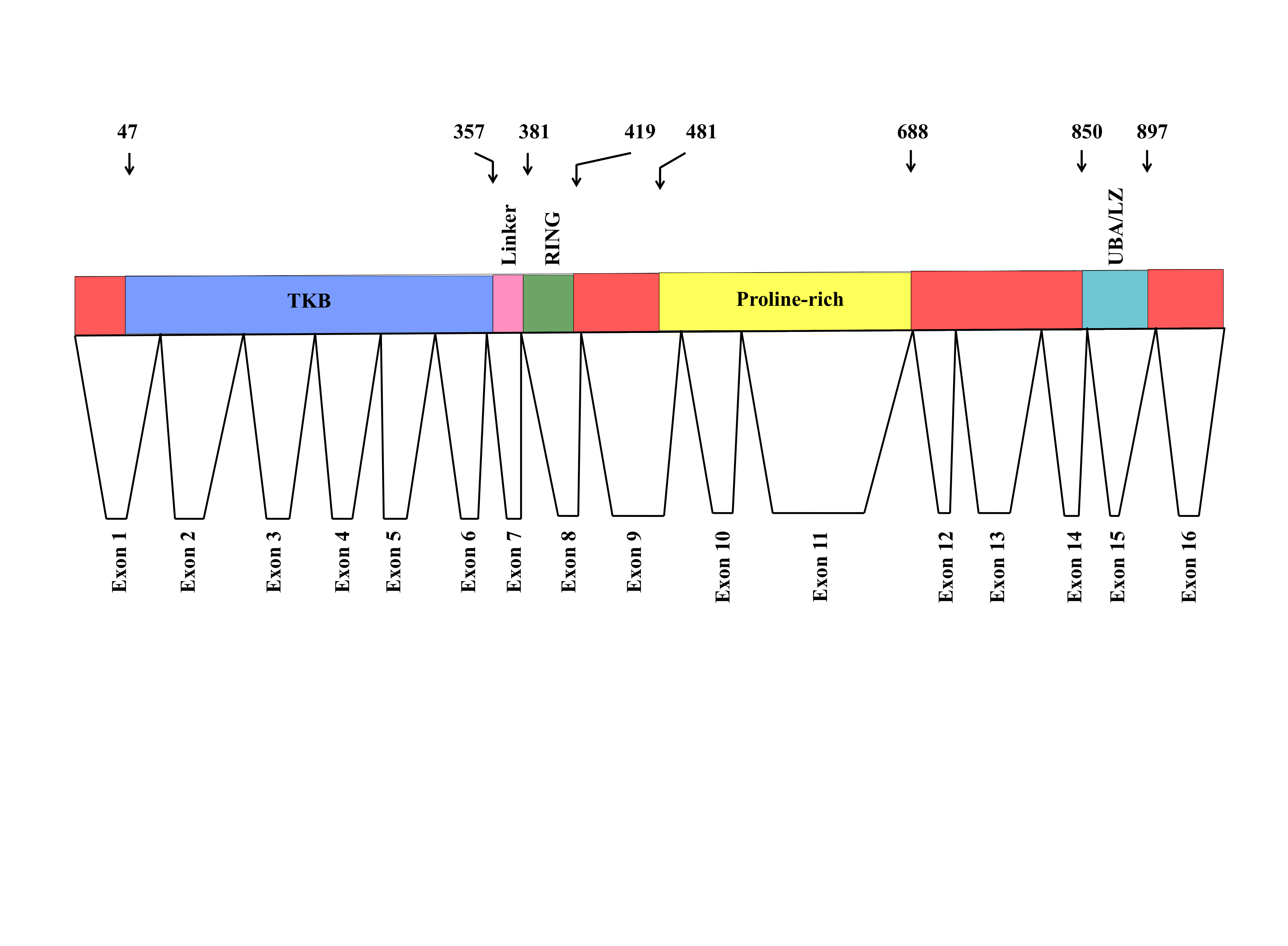

Supplement: Figure S1 — Schematic of c-CBL domains and associated exons. Numbers at the top indicate amino acid residues flanking the domains. (0.42 MB TIF) [file pone.0008972.s001.tif]

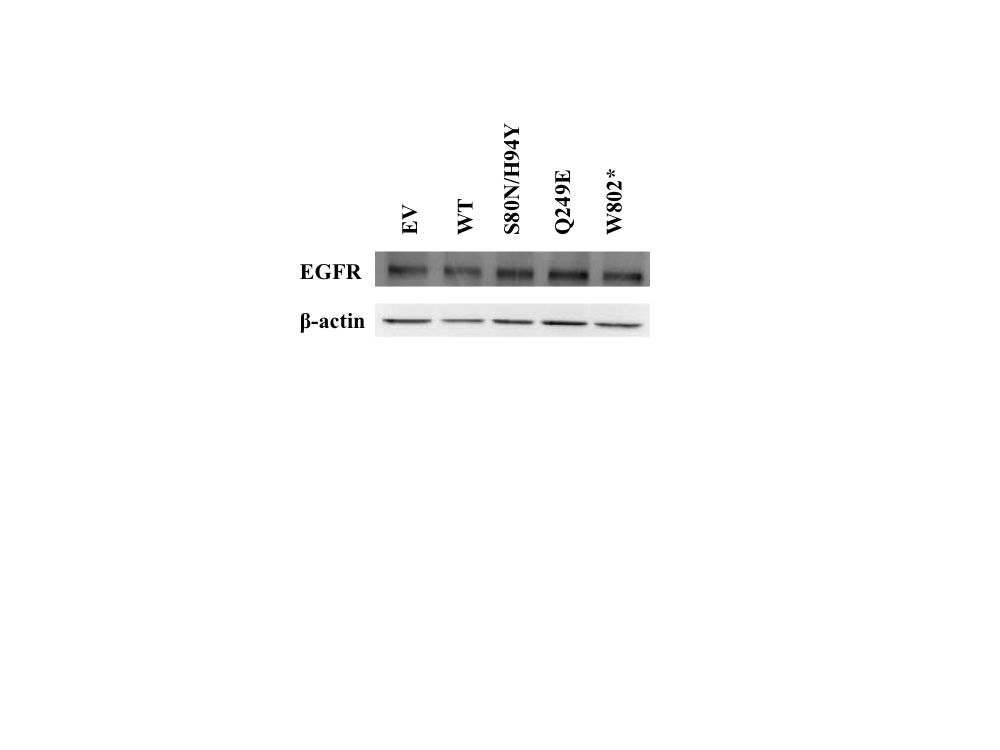

Supplement: Figure S2 — Western blot showing EGFR levels in different c-CBL mutants used in the study. EGFR levels were relatively unchanged among the three mutants compared to wild-type c-CBL. β-actin was used as a loading control. (3.00 MB TIF) [file pone.0008972.s002.tif]
