# Supplementary material for: CBL Is Frequently Altered in Lung Cancers: Its Relationship to Mutations in MET and EGFR Tyrosine Kinases
Source: PLoS One. 2010 Jan 29;5(1):e8972. doi: 10.1371/journal.pone.0008972 (PMC2813301; doi:10.1371/journal.pone.0008972)
Supplement: Table S1 — Number of samples analyzed in patients by race and sub-type of lung cancer. (0.03 MB DOC) [file pone.0008972.s003.doc]

**Supplementary Table 1. Number of samples analyzed in patients by race and sub-type of lung cancer.**

| **Race** | **Adenocarcinoma** | **Squamous cell carcinoma** | **Large cell carcinoma** | **Total** |
| --- | --- | --- | --- | --- |
| **Caucasian** | 20 | 10 | 20 | 50 |
| **African-American** | 10 | 10 | 9 | 29 |
| **Taiwanese** | 23 | 12 | 5 | 40 |
