# Supplementary material for: CBL Is Frequently Altered in Lung Cancers: Its Relationship to Mutations in MET and EGFR Tyrosine Kinases
Source: PLoS One. 2010 Jan 29;5(1):e8972. doi: 10.1371/journal.pone.0008972 (PMC2813301; doi:10.1371/journal.pone.0008972)
Supplement: Table S2 — c-Cbl PCR amplification primers. (0.04 MB DOC) [file pone.0008972.s004.doc]

**Supplementary Table 2. *c-Cbl* PCR amplification primers.**

| **Exon** | **Primers (5’🡪3’)** | **Amplicon (bp)** |
| --- | --- | --- |
| 2 | F: TAAAATGGTTGCCTGTGGGCAATG  R: TGTGTTACCCATTCAGGCAGTC | 477 |
| 3 | F: CATCTTGTATGGTGAATTTGGTGC  R: GACTCCGTCTCAAAAAGAAACCAC | 495 |
| 4 | F: GCTTAATGTGGCTCTCCTTCC  R: GTGAGGAGAAGAAAGCAGTTGG | 486 |
| 5 | F: CTCTGAGTTGGTTGTACATCTGAC  R: CAGAACCTTGGCTATTGCGAAAC | 290 |
| 6 | F: GTCTGTATCTTGCCTTGCCTTC  R: CCTAAGTTCCCAGACTCTAACAGATG | 241 |
| 7 and 8 | F: CTTACACCACGTTGCCCTTTTAG  R: CCTTGTATCAGTAAAGGCTATATAATACC | 736 |
| 9 | F: CGGTATTATATAGCCTTTACTGATACAAGG  R: CCAGTCTCCTAAACTGCCATCTTAC | 389 |
| 10 and 11 | F: CCTAGGTCTGGCCCATTTGTAG  R: CTGGCCCACACATATTTCTTAACAG | 775 |
| 12 | F: CAGAGGCTCAGCTGTGGTAAG  R: CAGAGCAATGAAACAGATGGCAG | 421 |
| 13 and 14 | F: GCTCTGTTCAATTTGAGTTATGTCTG  R: GCTTAGATCAAGCTATCTCAATTGCC | 759 |
| 15 | F: GTTTGGCCCACAGTAGACAATC  R: CTTGGGACTTTCCTCCCATTTAGAC | 467 |
| 16 | F: CTTGTGACTGAAGAGCACATGTAC  R: CTAGGTGCCACTTGAGTAATAACTC | 487 |
