# Supplementary material for: CBL Is Frequently Altered in Lung Cancers: Its Relationship to Mutations in MET and EGFR Tyrosine Kinases
Source: PLoS One. 2010 Jan 29;5(1):e8972. doi: 10.1371/journal.pone.0008972 (PMC2813301; doi:10.1371/journal.pone.0008972)
Supplement: Table S3 — Microsatellites and primer information. (0.03 MB DOC) [file pone.0008972.s005.doc]

**Supplementary Table 3. Microsatellites and primer information.**

| **Marker** | **Primers (5’🡪3’)** | **Microsatellite Location** | **Amplicon (bp)** |
| --- | --- | --- | --- |
| **D11S4628**  *(11q23)* | F: ATCTGGGGAGTGATTACCCC  R: GGCTGTTGCTTGGACTTCTC | Chr11:118,469,284-118,469,425 | 142 |
| **D11S1941E**  *(within CBL)* | F:TCCCGAGTTGAGGTAGAGTG R:GGCTGTTAGAAGTTGATGGC | Chr11:118,677,662-118,677,740 | 78 |
| **D11S4129**  *(11q23)* | F: GGCCACTGCCCTTACCATCA  R: ACAGCGACCACATCTCCTGC | Chr11: 118,899,756-118,899,858 | 109-119 |
| **D11S929**  *(11p control)* | F: CCCAGTTGCCGAACTACC  R: AGGCCCTTCCAAGATCAG | Chr11: 25,808,628-25,808,859 | 218-240 |
| **D11S1344**  *(11p control)* | F: CCCTGAACTTCTGCATTCAC  R: GCGCCTGGCTTGTACATATA | Chr11: 46,123,506-46,123,786 | 273-293 |
