# Supplementary material for: CBL Is Frequently Altered in Lung Cancers: Its Relationship to Mutations in MET and EGFR Tyrosine Kinases
Source: PLoS One. 2010 Jan 29;5(1):e8972. doi: 10.1371/journal.pone.0008972 (PMC2813301; doi:10.1371/journal.pone.0008972)
Supplement: Table S4 — Multiplex primer sets for LOH analysis. (0.03 MB DOC) [file pone.0008972.s006.doc]

**Supplementary Table 4. Multiplex primer sets for LOH analysis.**

| **Primer Set** | **Primers** | **Dyes** | **Dye Color** |
| --- | --- | --- | --- |
| **1** | D11S4129, D11S1344, D11S929 | VIC, 6FAM, PET | Green, Blue, Red |
| **2** | D11S1941E, D11S4628, D11S929 | 6FAM, NED, PET | Blue, Black, Red |
